# Diabetes and further risk of cancer: a nationwide population-based study

**DOI:** 10.1186/s12916-024-03430-y

**Published:** 2024-05-29

**Authors:** Wei-Chuan Chang, Tsung-Cheng Hsieh, Wen-Lin Hsu, Fang-Ling Chang, Hou-Ren Tsai, Ming-Shan He

**Affiliations:** 1 Department of Medical Research, Buddhist Tzu Chi General Hospital, Hualien, Taiwan; 2 Institute of Medical Sciences, Tzu Chi University, Hualien, Taiwan; 3 School of Medicine, Tzu Chi University, Hualien, Taiwan; 4 Department of Radiation Oncology, Buddhist Tzu Chi General Hospital, Hualien, Taiwan; 5 Department of Ophthalmology, Buddhist Tzu Chi General Hospital, No. 707, Sec. 3 Chung-Yung Road, Hualien, 970 Taiwan; 6 Department of Ophthalmology and Visual Science, Tzu Chi University, Hualien, Taiwan

**Keywords:** Diabetes, Cancer, Diabetic retinopathy, Proliferative diabetic retinopathy

## Abstract

**Background:**

Individuals with diabetes have a significantly higher risk of developing various forms of cancer, and the potential biological links between these two diseases are not completely understood.

**Methods:**

This was a longitudinal retrospective nationwide cohort study, a study design that allows us to examine the natural course of cancer development over an extended period of time with a large sample size. Initially, 3,111,975 and 22,208,395 eligible patients aged ≥ 20 years with and without diabetes, respectively, were matched by age, sex, and the Charlson comorbidity index. Ultimately, 1,751,457 patients were selected from each group. Stratified populations for diabetic retinopathy (DR) (*n* = 380,822) and without DR (*n* = 380,822) as well as proliferative DR (PDR) (*n* = 141,150) and non-proliferative DR (NPDR) (*n* = 141,150) were analyzed in this study. The main outcome measure was the first-time diagnosis of cancer during the follow-up period.

**Results:**

We observed a 20% higher risk of total cancer incidence [hazard ratios (HR), 1.20; *p* < 0.001] in the diabetes cohort compared to the non-diabetes cohort. The highest HR was observed for liver and pancreas cancers. Moderately increased risks were observed for oral, colon, gallbladder, reproductive (female), kidney, and brain cancer. Furthermore, there was a borderline significantly increased risk of stomach, skin, soft tissue, female breast, and urinary tract (except kidney) cancers and lymphatic and hematopoietic malignancies. The stratified analysis revealed that the total cancer incidence was significantly higher in the DR cohort compared to the non-DR cohort (HR, 1.31; *p* < 0.001), and there was a borderline increased risk in the PDR cohort compared to the NPDR cohort (HR, 1.13; *p* = 0.001).

**Conclusions:**

This study provides large-scale, nationwide, population-based evidence that diabetes is independently associated with an increased risk of subsequent development of total cancer and cancer at specific sites. Notably, this risk may further increase when DR develops.

## Background

The increasing global prevalence of diabetes and cancer has significant global health implications. Epidemiological evidence suggests that people with diabetes are at a significantly higher risk of various cancers, including hepatic, pancreatic, endometrial, colorectal, bladder, and breast cancers, whereas male patients with diabetes have a lower prevalence of prostate cancer than those without diabetes [1]. Clinical evidence has indicated a positive association between cancer and concomitant abnormalities in glucose metabolism. However, the potential biological links between these diseases are not completely understood.

Diabetic retinopathy (DR) is the most common microvascular complication in patients with diabetes and the leading global cause of vision loss in working middle-aged adults [2]. The pathological processes of DR include hyperglycemia and the polyol pathway, advanced glycation end-product formation, protein kinase C activation, hexosamine pathway flux, and poly (ADP-ribose) polymerase activation, which share similar pathogenic features with cancer initiation and progression [3–7]. Furthermore, oxidative stress, inflammation, vascular abnormalities, and angiogenesis are closely associated with pathological changes in the progression of DR, which are also involved in pathophysiological conditions for cancer development [8–10]. These findings suggest that DR and cancer may share similar pathogenic features and that improving diabetes control may further reduce the risk of cancer development.

Given the similarities in the pathogenesis and global impact of and mortality caused by both diseases, additional large-scale longitudinal studies that stratify diabetes into DR and non-DR subtypes and focus on the relationship between cancer and diabetes may help clarify the potential biological links between the two diseases. Therefore, this retrospective nationwide cohort study used data from the Taiwan National Health Insurance Research Database (NHIRD) to investigate the relationship between cancer and diabetes or DR. To achieve this goal, Cox proportional hazards regression analyses were performed using two cohorts with propensity-matching by age, sex, and comorbidities, which minimized confounding variables arising from the use of observational data.

## Methods

### Data sources

This nationwide, 1:1 matched, retrospective cohort study was conducted between January 2007 and December 2018. The database contains all registry files and details regarding original claims data obtained from the NHIRD, the Taiwan Cancer Registry (TCR), and the National Death Registry (NDR) of Taiwan. Taiwan launched a single-payer National Health Insurance program on March 1, 1995. As of 2014, 99.9% of Taiwan’s population were enrolled. The database of this program contains registration files and original claim data for reimbursement. Starting in 2002, Taiwan’s National Health Research Institutes established and continue to maintain NHIRD for public research purposes. The NHIRD, collecting data from almost all medical facilities in Taiwan, is a large, powerful data source for approved medical research [11]. Approximately 27.22 million individuals were included in this registry. All data in the database were encrypted to protect the privacy of individuals. The database provides detailed outpatient and inpatient claims data, including patient identification number, birth date, sex, treatment information, dates of admission and discharge, date of death, and diagnostic codes according to the International Classification of Diseases, 9th Revision, Clinical Modification (ICD-9-CM) codes before 2016 and ICD-10-CM codes (10th revision) since 2016. Each patient has a unique encrypted identifier that can be linked to the TCR and NDR. All datasets were interlinked using patient identification numbers.

### Study cohort and patient selection

**Fig. 1:**
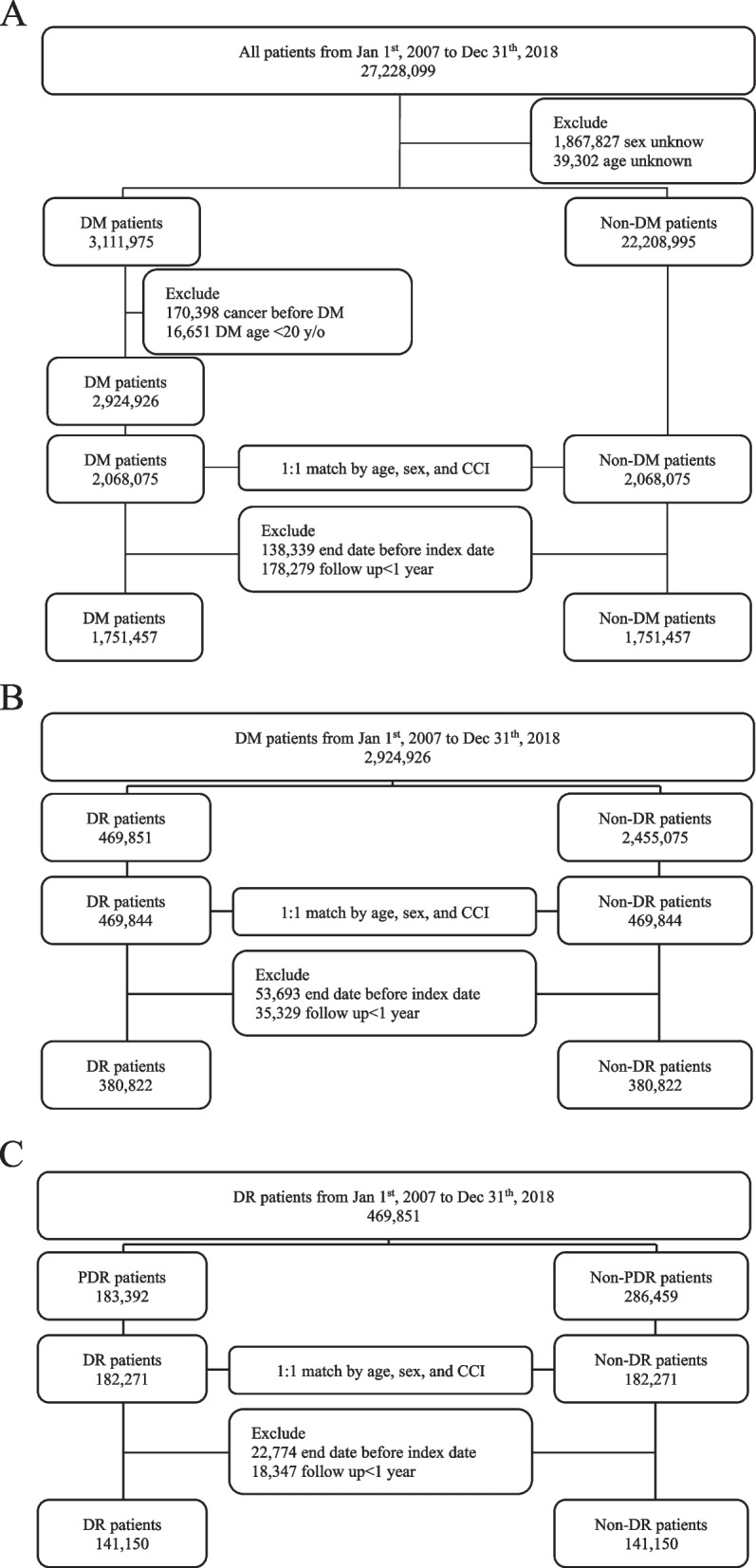
Study protocol and profile. **A** The selection flow chart and selected populations for the diabetes group and the control cohort. **B** The stratification flow chart and the stratified populations for diabetes with DR vs. diabetes without DR. **C** The stratification flow chart and the stratified populations for PDR vs. non-PDR. DM, diabetes mellitus; DR, diabetic retinopathy; PDR, proliferative diabetic retinopathy; y/o, years old

This study was approved by the Institutional Review Board of Tzu Chi Hospital, Hualien (TCHIRB109-108-C). For this retrospective study, informed consent was waived in accordance with the institutional guidelines. Among a total of 27,228,099 patients in the NHIRD between January 1, 2007, and December 31, 2018, those with unknown sex (*n* = 1,867,827) and age (*n* = 39,302) were excluded; the exclusion of these cases was based on the rationale that sex and age are two major variables for propensity matching in this study. Overall, 3,111,975 patients with primary diabetes (ICD-9-CM 250 or ICD-10-CM E10, E11) and 22,208,395 patients without diabetes were initially enrolled in the diabetes and non-diabetes groups, respectively, as the main study cohorts. Patients with secondary diabetes caused by factors that may also be independent risk factors for cancer (e.g., certain viral infections like hepatitis B virus or C virus) were excluded. Participants were further excluded from the diabetes group if they had cancer before the diagnosis of diabetes (*n* = 170,398) or if they were aged < 20 years (*n* = 16,651). To avoid confounding effects of patients’ characteristics and comorbidities, the resulting available patients with and without diabetes were further matched in a 1:1 ratio by age, sex, and Charlson index comorbidity (CCI). Finally, 2,068,075 patients from each group were included in this study. The index date was defined as the date of diabetes onset. After excluding the end date before the index date (*n* = 138,339) and follow-up of less than 1 year (*n* = 178,279), 1,751,457 paired study participants in each group were obtained. In addition to the main study cohort, stratified populations for diabetes with DR (ICD-9-CM 362.0X or ICD-10-CM E10.3X, E11.3X; *n* = 380,822) and without DR (*n* = 380,822) were obtained. Patients with DR were further stratified into proliferative DR (PDR, *n* = 141,150) and non-PDR (NPDR, *n* = 141,150) groups according to the presence (ICD-9-CM 362.02 or ICD-10-CM E10.35X, E11.35X) or absence (ICD-9-CM 362.01 or ICD-10-CM E10.32X, E10.33X, E10.34X, E11.32X, E11.33X, E11.34X) of retinal neovascularization. All stratifications were performed using similar exclusion criteria and matching procedures to those of the main cohorts. The detailed data flow of the study is shown in Fig. 1.

### Outcomes measures

The study endpoint was the first incidence of cancer at any site during follow-up, identified according to TCR. Only the first occurrence of cancer was considered when calculating the cancer incidence. As identified by the corresponding ICD-10-CM codes of cancer, the sites were defined as follows: the lip, oral cavity, and pharynx (C00–C14); digestive organs (C15–C26), including the esophagus (C15), stomach (C16), colon (C18), liver (C22), gallbladder (C23), and pancreas (C25); respiratory and intrathoracic organs (C30–C39); the bone and articular cartilage (C40–C41); the skin (C43–C44); the soft tissue (C45–C49); the breast (C50); female genital organs (C51–C58); male genital organs (C60–C63); the urinary tract (C64–C68), including the kidney (C64) and others (C65–68); the eye (C69); the brain and other parts of the central nervous system (C70–C72); and lymphoid, hematopoietic, and related tissue (C81–C96). If a participant had lesions of different severity levels in both eyes, the grade assigned to them was that of the more severely involved eye. All outcomes were assessed during the follow-up period between the index date and December 31, 2018. Baseline comorbidities were identified using the ICD-9 codes, including CCI, hypertension (401. X–405.X, 437.2, 362.11), and hyperlipidemia (272.X).

### Statistical analysis

Baseline characteristics, including age, sex, hypertension, hyperlipidemia, and CCI score were compared between two study groups using standardized mean difference (SMD). The incidence rate of cancer was calculated per 100,000 person-years, and the incidence ratio between two study groups was calculated. The Cox proportional hazards model was used to assess the adjusted hazard ratios (HR) and 99% confidence intervals (CI). The classification for the increased cancer risk was defined as follows: borderline, HR between 1.10 and 1.19; moderate, HR between 1.20 and 1.49; and high, HR ≥ 1.50. All models were adjusted for the characteristics listed in Table 1. Data analyses were performed using SAS version 9.4 for Windows (SAS Institute, Inc., Cary, NC, USA). All statistical tests were 2-sided, and a *p*-value < 0.01 or SMD > 0.1 was considered statistically significant.

**Table 1:** Characteristics of the study population

			Diabetes vs. non-diabetes			Diabetes w/DR vs. diabetes w/o DR			PDR vs. NPDR		
Variable	Category	Statistic	Diabetes *n* = 1,751,457	Non-diabetes *n* = 1,751,457	Standard diff	Diabetes w/DR *n* = 380,822	Diabetes w/o DR *n* = 380,822	Standard diff	PDR *n* = 141,150	NPDR *n* = 141,150	Standard diff
Sex	Female	*n* (%)	868,837 (49.61%)	868,837 (49.61%)	< 0.001	198,408 (52.10%)	198,408 (52.10%)	< 0.001	72,117 (51.09%)	72,117 (51.09%)	< 0.001
	Male	*n* (%)	882,620 (50.39%)	882,620 (50.39%)		182,414 (47.90%)	182,414 (47.90%)		69,033 (48.91%)	69,033 (48.91%)	
Age, years		Mean ± SD	57.80 ± 13.01	57.80 ± 13.01	< 0.001	61.08 ± 11.34	61.08 ± 11.34	< 0.001	60.31 ± 11.12	60.31 ± 11.12	< 0.001
CCI total		Mean ± SD	0.22 ± 0.70	0.22 ± 0.70	< 0.001	0.43 ± 0.89	0.43 ± 0.89	< 0.001	0.40 ± 0.85	0.40 ± 0.85	< 0.001
Hypertension		*n* (%)	524,891 (29.97%)	152,951 (8.73%)	0.559	76,081 (19.98%)	147,750 (38.80%)	0.422	22,552 (15.98%)	32,188 (22.80%)	0.173
Hyperlipidemia		*n* (%)	583,458 (33.31%)	70,932 (4.05%)	0.808	112,016 (29.41%)	135,058 (35.46%)	0.131	35,391 (25.07%)	46,366 (32.85%)	0.173

*Diff *Difference, *w/o *Without, *w/ *With

## Results

The demographic characteristics and comorbidities of all cohorts are shown in Table 1.

### Diabetes versus non-diabetes

During the 12-year follow-up period, the overall mean annual incidence of total cancer was higher in patients with diabetes than patients without (1309.74 per 100,000 person-years vs. 1130.13 per 100,000 person-years; incidence ratio, 1.17) (Table 2).

**Table 2:** Incidence of events (100,000 person-years)

	Diabetes vs. non-diabetes							Diabetes w/DR vs. diabetes w/o DR							PDR vs. NPDR						
Outcome	Diabetes			Non-diabetes			Ratio	Diabetes w/DR			Diabetes w/o DR			Ratio	PDR			NPDR			Ratio
Cancer	PY	Event	Rate	PY	Event	Rate		PY	Event	Rate	PY	Event	Rate		PY	Event	Rate	PY	Event	Rate	
**All sites**	13,833,642	181,184	1309.74	14,356,438	162,246	1130.13	1.17 (*p* < 0.001)	2,552,252	38,139	1494.33	2,731,208	31,450	1151.51	1.32 (*p* < 0.001)	880,079.10	12,890	1464.64	981,677.50	13,052	1329.56	1.13 (*p* < 0.001)
**Lip, oral cavity, and pharynx**	13,833,642	7993	57.78	14,356,438	6799	47.36	1.21 (*p* < 0.001)	2,552,252	1361	53.33	2,731,208	939	34.38	1.52 (*p* < 0.001)	880,079.10	484	55	981,677.50	464	47.27	1.14 (*p* = 0.040)
**Digestive organs**	13,833,642	49,755	359.67	14,356,438	40,751	283.85	1.25 (*p* < 0.001)	2,552,252	10,355	405.72	2,731,208	7079	259.19	1.55 (*p* < 0.001)	880,079.10	3620	411.33	981,677.50	3182	324.14	1.25 (*p* < 0.001)
Esophagus	13,833,642	1484	10.73	14,356,438	2024	14.1	0.75 (*p* < 0.001)	2,552,252	249	9.76	2,731,208	208	7.62	1.26 (*p* = 0.013)	880,079.10	82	9.32	981,677.50	85	8.66	1.06 (*p* = 0.691)
Stomach	13,833,642	5038	36.42	14,356,438	4701	32.74	1.10 (*p* < 0.001)	2,552,252	940	36.83	2,731,208	706	25.85	1.41 (*p* < 0.001)	880,079.10	356	40.45	981,677.50	270	27.5	1.45 (*p* < 0.001)
Colon	13,833,642	19,679	142.25	14,356,438	16,672	116.13	1.21 (*p* < 0.001)	2,552,252	3724	145.91	2,731,208	2835	103.8	1.39 (*p* < 0.001)	880,079.10	1284	145.9	981,677.50	1229	125.19	1.15 (*p* < 0.001)
Liver	13,833,642	17,684	127.83	14,356,438	13,290	92.57	1.37 (*p* < 0.001)	2,552,252	4313	168.99	2,731,208	2417	88.5	1.88 (*p* < 0.001)	880,079.10	1522	172.94	981,677.50	1255	127.84	1.33 (*p* < 0.001)
Gallbladder	13,833,642	1424	10.29	14,356,438	1157	8.06	1.27 (*p* < 0.001)	2,552,252	273	10.7	2,731,208	226	8.27	1.28 (*p* = 0.006)	880,079.10	101	11.48	981,677.50	74	7.54	1.50 (*p* = 0.009)
Pancreas	13,833,642	3437	24.85	14,356,438	1994	13.89	1.77 (*p* < 0.001)	2,552,252	666	26.09	2,731,208	549	20.1	1.28 (*p* < 0.001)	880,079.10	214	24.32	981,677.50	219	22.31	1.08 (*p* = 0.428)
**Respiratory and intrathoracic organs**	13,833,642	16,844	121.76	14,356,438	17,615	122.7	0.98 (*p* = 0.124)	2,552,252	3224	126.32	2,731,208	2447	89.59	1.39 (*p* < 0.001)	880,079.10	1066	121.13	981,677.50	1051	107.06	1.12 (*p* = 0.011)
**Bone and articular cartilage**	13,833,642	293	2.12	14,356,438	246	1.71	1.22 (*p* = 0.021)	2,552,252	58	2.27	2,731,208	36	1.32	1.70 (*p* = 0.012)	880,079.10	20	2.27	981,677.50	15	1.53	1.46 (*p* = 0.271)
**Skin**	13,833,642	3660	26.46	14,356,438	3268	22.76	1.15 (*p* < 0.001)	2,552,252	717	28.09	2,731,208	554	20.28	1.37 (*p* < 0.001)	880,079.10		27.276	981,677.50	229	23.33	1.16 (*p* = 0.112)
**Mesothelial and soft tissue**	13,833,642	766	5.54	14,356,438	685	4.77	1.15 (*p* = 0.008)	2,552,252	149	5.84	2,731,208	102	3.73	1.55 (*p* < 0.001)	880,079.10	48	5.45	981,677.50	54	5.5	0.99 (*p* = 0.949)
**Female breast**	7,005,260	8196	117	7,292,408	7239	99.27	1.16 (*p* < 0.001)	1,371,715	1417	103.3	1,458,490	1152	78.99	1.29 (*p* < 0.001)	465,678.30	427	91.69	514,085.90	514	99.98	0.90 (*p* = 0.111)
**Female genital**	13,833,642	4993	36.09	14,356,438	4168	29.03	1.23 (*p* < 0.001)	2,552,252	851	33.34	2,731,208	689	25.23	1.30 (*p* < 0.001)	880,079.10	328	37.27	981,677.50	268	27.3	1.34 (*p* < 0.001)
**Male genital**	13,833,642	7921	57.26	14,356,438	8107	56.47	1.00 (*p* = 0.806)	2,552,252	1248	48.9	2,731,208	1236	45.25	1.07 (*p* = 0.104)	880,079.10	338	38.41	981,677.50	526	53.58	0.71 (*p* < 0.001)
**Urinary tract**	13,833,642	8246	59.61	14,356,438	6671	46.47	1.27 (*p* < 0.001)	2,552,252	1740	68.18	2,731,208	1147	42	1.60 (*p* < 0.001)	880,079.10	664	75.45	981,677.50	529	53.89	1.38 (*p* < 0.001)
Kidney	13,833,642	4300	31.08	14,356,438	3101	21.6	1.43 (*p* < 0.001)	2,552,252	919	36.01	2,731,208	581	21.27	1.67 (*p* < 0.001)	880,079.10	369	41.93	981,677.50	271	27.61	1.50 (*p* < 0.001)
Other than kidney	13,833,642	3946	28.52	14,356,438	3570	24.87	1.14 (*p* < 0.001)	2,552,252	821	32.17	2,731,208	566	20.72	1.53 (*p* < 0.001)	880,079.10	295	33.52	981,677.50	258	26.28	1.26 (*p* = 0.008)
**Eye**	13,833,642	134	0.97	14,356,438	138	0.96	1.00 (*p* = 0.967)	2,552,252	22	0.86	2,731,208	18	0.66	1.28 (*p* = 0.444)	880,079.10	4	0.45	981,677.50	12	1.22	0.36 (*p* = 0.078)
**Brain and other central nervous system**	13,833,642	1264	9.14	14,356,438	1045	7.28	1.24 (*p* < 0.001)	2,552,252	213	8.35	2,731,208	174	6.37	1.29 (*p* = 0.012)	880,079.10	63	7.16	981,677.50	64	6.52	1.08 (*p* = 0.680)
**Lymph and hematopoietic**	13,833,642	4925	35.6	14,356,438	4608	32.1	1.10 (*p* < 0.001)	2,552,252	998	39.1	2,731,208	711	26.03	1.48 (*p* < 0.001)	880,079.10	339	38.52	981,677.50	333	33.92	1.12 (*p* = 0.148)

*Event*, number of events; *PY*, total person-years; *Rate*, incidence; *Ratio*, incidence ratio; *w/o*, without; *w/*, with. The incidence rate (100,000 person-years) was the incidence (event) divided by the total person-years. *Statistically significant at *p* < 0.01

In the multivariate survival analysis, patients with diabetes (HR, 1.20; 99% CI: 1.19–1.21; *p* < 0.001) and CCI (HR. 1.23; 99% CI: 1.22–1.24; *p* < 0.001) showed moderately increased risk of subsequent total cancer development; male sex (HR, 1.19; 99% CI: 1.18–1.20; *p* < 0.001) and hypertension (HR, 1.10; 99% CI: 1.09–1.11; *p* < 0.001) both had a borderline significantly higher incidence of subsequent total cancer, except for hyperlipidemia (HR, 0.86; 99% CI: 0.85–0.87; *p* < 0.001), which was independently associated with a decreased risk of subsequent total cancer.

Patients with diabetes had a significantly higher incidence of subsequent liver (HR, 1.69; 99% CI: 1.63–1.74; *p* < 0.001) and pancreas (HR, 1.87; 99% CI: 1.73–2.02; *p* < 0.001) cancers. We also observed a moderately increased risk of the oral cavity and pharynx (HR, 1.30; 99% CI: 1.24–1.36; *p* < 0.001), colon (HR, 1.25; 99% CI: 1.21–1.29; *p* < 0.001), gallbladder (HR, 1.34; 99% CI: 1.20–1.50; *p* < 0.001), female genital organs (HR, 1.30; 99% CI: 1.22–1.37; *p* < 0.001), kidney (HR, 1.44; 99% CI: 1.34–1.53; *p* < 0.001), and brain and other parts of central nervous system cancers (HR, 1.31; 99% CI: 1.17–1.48; *p* < 0.001). Furthermore, there were borderline increases in the risk of stomach (HR, 1.19; 99% CI: 1.13–1.26; *p* < 0.001), skin (HR,1.17; 99% CI: 1.09–1.25; *p* < 0.001), mesothelial and soft tissue (HR, 1.18; 99% CI: 1.02–1.37; *p* = 0.003), female breast (HR, 1.17; 99% CI: 1.11–1.22; *p* < 0.001), and urinary tract cancer (except kidney) (HR, 1.17; 99% CI: 1.10–1.25; *p* < 0.001) and lymphatic and hematopoietic malignancies (HR, 1.19; 99% CI, 1.13–1.26; *p* < 0.001). Conversely, patients with diabetes had a lower risk of subsequent esophagus cancer than those without diabetes (HR, 0.83; 99% CI: 0.76–0.92; *p* < 0.001) (Table 3).

**Table 3:** Predictors of total cancer and cancer in specific sites by multivariate analysis

		Diabetes vs. non-diabetes		Diabetes w/DR vs. diabetes w/o DR		PDR vs. NPDR	
Outcome	Effect	HR (99% CI)	*p*-value	HR (99% CI)	*p*-value	HR (99% CI)	*p*-value
**All sites**	Group	1.20 (1.19–1.21)	*p* < 0.001	1.31 (1.28–1.34)	*p* < 0.001	1.13 (1.10–1.17)	*p* < 0.001
	Sex (ref.: female)	1.19 (1.18–1.20)	*p* < 0.001	1.25 (1.23–1.28)	*p* < 0.001	1.19 (1.16–1.23)	*p* < 0.001
	CCI	1.23 (1.22–1.24)	*p* < 0.001	1.11 (1.10–1.13)	*p* < 0.001	1.12 (1.11–1.14)	*p* < 0.001
	HTN	1.10 (1.09–1.11)	*p* < 0.001	0.97 (0.94–0.99)	*p* < 0.001	1.01 (0.97–1.06)	*p* = 0.370
	HLD	0.86 (0.85–0.87)	*p* < 0.001	0.92 (0.90–0.94)	*p* < 0.001	0.91 (0.88–0.94)	*p* < 0.001
**Lip, oral cavity and pharynx**	Group	1.30 (1.24–1.36)	*p* < 0.001	1.46 (1.30–1.63)	*p* < 0.001	1.15 (0.97–1.36)	*p* = 0.036
	Sex (ref.: female)	4.58 (4.34–4.84)	*p* < 0.001	4.31 (3.78–4.92)	*p* < 0.001	3.88 (3.17–4.76)	*p* < 0.001
	CCI	1.14 (1.11–1.18)	*p* < 0.001	1.04 (0.98–1.10)	*p* = 0.089	1.11 (1.02–1.22)	*p* = 0.002
	HTN	0.79 (0.75–0.84)	*p* < 0.001	0.77 (0.68–0.89)	*p* < 0.001	0.92 (0.73–1.15)	*p* = 0.312
	HLD	0.90 (0.84–0.95)	*p* < 0.001	0.96 (0.85–1.07)	*p* = 0.311	0.99 (0.82–1.19)	*p* = 0.865
**Digestive organs**	Group	1.40 (1.37–1.42)	*p* < 0.001	1.47 (1.41–1.53)	*p* < 0.001	1.23 (1.15–1.31)	*p* < 0.001
	Sex (ref.: female)	1.35 (1.33–1.37)	*p* < 0.001	1.36 (1.31–1.41)	*p* < 0.001	1.32 (1.24–1.40)	*p* < 0.001
	CCI	1.31 (1.30–1.33)	*p* < 0.001	1.20 (1.17–1.22)	*p* < 0.001	1.23 (1.20–1.27)	*p* < 0.001
	HTN	0.91 (0.89–0.94)	*p* < 0.001	0.82 (0.78–0.86)	*p* < 0.001	0.93 (0.86–1.01)	*p* = 0.020
	HLD	0.71 (0.70–0.73)	*p* < 0.001	0.74 (0.71–0.78)	*p* < 0.001	0.76 (0.70–0.81)	*p* < 0.001
Esophagus	Group	0.83 (0.76–0.92)	*p* < 0.001	1.22 (0.95–1.56)	*p* = 0.042	1.09 (0.73–1.63)	*p* = 0.598
	Sex (ref.: female)	6.73 (5.92–7.66)	*p* < 0.001	4.53 (3.35–6.13)	*p* < 0.001	4.13 (2.53–6.74)	*p* < 0.001
	CCI	1.18 (1.11–1.24)	*p* < 0.001	1.07 (0.94–1.22)	*p* = 0.163	1.16 (0.95–1.42)	*p* = 0.053
	HTN	0.88 (0.77–1.01)	*p* = 0.117	0.84 (0.62–1.12)	*p* = 0.115	1.17 (0.72–1.92)	*p* = 0.408
	HLD	0.75 (0.65–0.86)	*p* < 0.001	0.85 (0.65–1.11)	*p* = 0.122	0.92 (0.58–1.45)	*p* = 0.626
Stomach	Group	1.19 (1.13–1.26)	*p* < 0.001	1.37 (1.20–1.57)	*p* < 0.001	1.44 (1.16–1.77)	*p* < 0.001
	Sex (ref.: female)	1.38 (1.31–1.45)	*p* < 0.001	1.35 (1.19–1.54)	*p* < 0.001	1.28 (1.04–1.57)	*p* = 0.002
	CCI	1.21 (1.18–1.25)	*p* < 0.001	1.04 (0.97–1.12)	*p* = 0.176	1.13 (1.01–1.26)	*p* = 0.004
	HTN	1.01 (0.94–1.08)	*p* = 0.792	0.94 (0.81–1.09)	*p* = 0.256	1.10 (0.84–1.43)	*p* = 0.368
	HLD	0.75 (0.69–0.81)	*p* < 0.001	0.76 (0.66–0.88)	*p* < 0.001	0.72 (0.56–0.93)	*p* = 0.001
Colon	Group	1.25 (1.21–1.29)	*p* < 0.001	1.36 (1.27–1.45)	*p* < 0.001	1.15 (1.04–1.28)	*p* = 0.001
	Sex (ref.: female)	1.18 (1.15–1.22)	*p* < 0.001	1.31 (1.23–1.39)	*p* < 0.001	1.29 (1.17–1.43)	*p* < 0.001
	CCI	1.18 (1.16–1.20)	*p* < 0.001	1.02 (0.98–1.06)	*p* = 0.181	1.06 (1.00–1.13)	*p* = 0.007
	HTN	1.05 (1.01–1.09)	*p* = 0.001	0.92 (0.85–0.99)	*p* = 0.002	1.06 (0.93–1.21)	*p* = 0.261
	HLD	0.88 (0.85–0.91)	*p* < 0.001	0.94 (0.88–1.01)	*p* = 0.019	0.94 (0.83–1.05)	*p* = 0.143
Liver	Group	1.69 (1.63–1.74)	*p* < 0.001	1.71 (1.60–1.83)	*p* < 0.001	1.27 (1.15–1.40)	*p* < 0.001
	Sex (ref.: female)	1.51 (1.47–1.56)	*p* < 0.001	1.44 (1.35–1.54)	*p* < 0.001	1.38 (1.25–1.52)	*p* < 0.001
	CCI	1.51 (1.49–1.52)	*p* < 0.001	1.41 (1.38–1.45)	*p* < 0.001	1.42 (1.37–1.48)	*p* < 0.001
	HTN	0.76 (0.73–0.79)	*p* < 0.001	0.71 (0.66–0.77)	*p* < 0.001	0.76 (0.67–0.87)	*p* < 0.001
	HLD	0.49 (0.47–0.52)	*p* < 0.001	0.54 (0.50–0.59)	*p* < 0.001	0.58 (0.51–0.66)	*p* < 0.001
Gallbladder	Group	1.34 (1.20–1.50)	*p* < 0.001	1.22 (0.96–1.54)	*p* = 0.035	1.42 (0.96–2.12)	*p* = 0.022
	Sex (ref.: female)	0.84 (0.76–0.93)	*p* < 0.001	0.88 (0.70–1.11)	*p* = 0.152	0.81 (0.55–1.21)	*p* = 0.176
	CCI	1.23 (1.16–1.30)	*p* < 0.001	1.08 (0.95–1.22)	*p* = 0.137	1.00 (0.79–1.27)	*p* = 0.969
	HTN	0.93 (0.81–1.06)	*p* = 0.145	0.86 (0.65–1.13)	*p* = 0.143	0.91 (0.53–1.55)	*p* = 0.632
	HLD	0.87 (0.75–0.99)	*p* = 0.007	0.71 (0.54–0.93)	*p* = 0.001	0.53 (0.32–0.89)	*p* = 0.002
Pancreas	Group	1.87 (1.73–2.02)	*p* < 0.001	1.23 (1.06–1.44)	*p* < 0.001	1.09 (0.85–1.40)	*p* = 0.395
	Sex (ref.: female)	1.04 (0.97–1.11)	*p* = 0.176	1.02 (0.88–1.19)	*p* = 0.699	0.97 (0.76–1.25)	*p* = 0.784
	CCI	1.16 (1.12–1.22)	*p* < 0.001	1.05 (0.96–1.14)	*p* = 0.172	1.06 (0.93–1.22)	*p* = 0.253
	HTN	0.93 (0.85–1.02)	*p* = 0.057	0.87 (0.73–1.03)	*p* = 0.037	1.03 (0.75–1.48)	*p* = 0.784
	HLD	0.87 (0.80–0.96)	*p* < 0.001	0.84 (0.71–0.99)	*p* = 0.006	1.02 (0.78–1.34)	*p* = 0.851
**Respiratory and intrathoracic organs**	Group	1.04 (1.01–1.07)	*p* < 0.001	1.35 (1.26–1.45)	*p* < 0.001	1.11 (0.99–1.25)	*p* = 0.014
	Sex (ref.: female)	1.51 (1.46–1.55)	*p* < 0.001	1.60 (1.49–1.71)	*p* < 0.001	1.43 (1.27–1.60)	*p* < 0.001
	CCI	1.20 (1.18–1.22)	*p* < 0.001	1.09 (1.06–1.14)	*p* < 0.001	1.14 (1.08–1.21)	*p* < 0.001
	HTN	0.99 (0.96–1.03)	*p* = 0.625	0.88 (0.81–0.95)	*p* < 0.001	0.94 (0.81–1.09)	*p* = 0.295
	HLD	0.83 (0.80–0.87)	*p* < 0.001	0.87 (0.81–0.94)	*p* < 0.001	0.93 (0.82–1.06)	*p* = 0.147
**Bone and articular cartilage**	Group	1.21 (0.95–1.55)	*p* = 0.044	1.70 (0.97–2.98)	*p* = 0.015	1.58 (0.65–3.84)	*p* = 0.188
	Sex (ref.: female)	0.95 (0.76–1.19)	*p* = 0.573	1.00 (0.59–1.70)	*p* = 0.989	0.82 (0.34–1.97)	*p* = 0.550
	CCI	1.18 (1.03–1.34)	*p* = 0.001	1.14 (0.87–1.49)	*p* = 0.220	1.36 (0.95–1.96)	*p* = 0.028
	HTN	0.94 (0.69–1.26)	*p* = 0.569	1.01 (0.54–1.88)	*p* = 0.960	1.99 (0.77–5.18)	*p* = 0.063
	HLD	1.07 (0.80–1.44)	*p* = 0.544	0.89 (0.50–1.60)	*p* = 0.609	0.93 (0.35–2.46)	*p* = 0.846
**Skin**	Group	1.17 (1.09–1.25)	*p* < 0.001	1.33 (1.14–1.54)	*p* < 0.001	1.15 (0.90–1.46)	*p* = 0.144
	Sex (ref.: female)	0.99 (0.93–1.06)	*p* = 0.791	1.11 (0.96–1.28)	*p* = 0.069	1.09 (0.86–1.38)	*p* = 0.358
	CCI	1.25 (1.21–1.29)	*p* < 0.001	1.05 (0.97–1.14)	*p* = 0.111	1.11 (0.98–1.26)	*p* = 0.035
	HTN	1.22 (1.13–1.32)	*p* < 0.001	0.87 (0.73–1.04)	*p* = 0.041	0.96 (0.71–1.31)	*p* = 0.749
	HLD	0.84 (0.77–0.91)	*p* < 0.001	0.90 (0.77–1.05)	*p* = 0.081	0.860 (0.65–1.13)	*p* = 0.153
**Mesothelial and soft tissue**	Group	1.18 (1.02–1.37)	*p* = 0.003	1.56 (1.11–2.20)	*p* < 0.001	0.98 (0.58–1.63)	*p* = 0.901
	Sex (ref.: female)	1.20 (1.05–1.38)	*p* < 0.001	1.32 (0.95–1.83)	*p* = 0.029	1.36 (0.81–2.27)	*p* = 0.125
	CCI	1.17 (1.08–1.27)	*p* < 0.001	0.90 (0.72–1.11)	*p* = 0.181	0.88 (0.62–1.25)	*p* = 0.364
	HTN	0.97 (0.81–1.17)	*p* = 0.680	1.08 (0.74–1.57)	*p* = 0608	0.86 (0.43–1.73)	*p* = 0.577
	HLD	0.93 (0.77–1.11)	*p* = 0.283	0.93 (0.65–1.33)	*p* = 0.602	1.01 (0.58–1.78)	*p* = 0.950
**Female breast**	Group	1.17 (1.11–1.22)	*p* < 0.001	1.28 (1.15–1.42)	*p* < 0.001	0.91 (0.76–1.07)	*p* = 0.131
	CCI	1.10 (1.07–1.13)	*p* < 0.001	0.97 (0.91–1.03)	*p* = 0.157	1.08 (0.98–1.19)	*p* = 0.054
	HTN	0.90 (0.86–0.96)	*p* < 0.001	0.90 (0.80–1.02)	*p* = 0.025	0.95 (0.77–1.17)	*p* = 0.521
	HLD	1.06 (1.00–1.12)	*p* = 0.009	1.20 (1.08–1.33)	*p* < 0.001	1.11 (0.93–1.33)	*p* = 0.129
**Female genital**	Group	1.30 (1.22–1.37)	*p* < 0.001	1.25 (1.09–1.43)	*p* < 0.001	1.31 (1.06–1.62)	*p* = 0.001
	CCI	1.08 (1.04–1.12)	*p* < 0.001	0.96 (0.88–1.04)	*p* = 0.199	1.03 (0.91–1.17)	*p* = 0.554
	HTN	0.92 (0.86–0.99)	*p* = 0.002	0.86 (0.74–1.01)	*p* = 0.015	0.91 (0.69–1.20)	*p* = 0.367
	HLD	0.89 (0.82–0.95)	*p* < 0.001	0.94 (0.82–1.08)	*p* = 0.271	0.94 (0.74–1.19)	*p* = 0.501
**Male genital**	Group	0.96 (0.92–1.00)	*p* = 0.018	1.11 (0.99–1.23)	*p* = 0.012	0.74 (0.62–0.89)	*p* < 0.001
	CCI	1.19 (1.16–1.21)	*p* < 0.001	1.01 (0.96–1.07)	*p* = 0.625	1.01 (0.92–1.12)	*p* = 0.749
	HTN	1.28 (1.21–1.36)	*p* < 0.001	1.13 (1.00–1.27)	*p* = 0.008	1.42 (1.15–1.74)	*p* < 0.001
	HLD	0.99 (0.93–1.04)	*p* = 0.510	1.10 (0.98–1.23)	*p* = 0.029	1.06 (0.87–1.28)	*p* = 0.469
**Urinary tract**	Group	1.29 (1.24–1.36)	*p* < 0.001	1.59 (1.44–1.76)	*p* < 0.001	1.39 (1.19–1.62)	*p* < 0.001
	Sex (ref.: female)	1.28 (1.23–1.34)	*p* < 0.001	1.26 (1.14–1.38)	*p* < 0.001	1.26 (1.08–1.46)	*p* < 0.001
	CCI	1.25 (1.22–1.28)	*p* < 0.001	1.13 (1.08–1.19)	*p* < 0.001	1.23 (1.15–1.32)	*p* < 0.001
	HTN	1.07 (1.01–1.13)	*p* = 0.003	0.98 (0.87–1.09)	*p* = 0.568	0.98 (0.81–1.18)	*p* = 0.748
	HLD	0.91 (0.86–0.96)	*p* < 0.001	0.93 (0.83–1.03)	*p* = 0.056	0.99 (0.84–1.17)	*p* = 0.909
Kidney	Group	1.44 (1.34–1.53)	*p* < 0.001	1.65 (1.44–1.90)	*p* < 0.001	1.50 (1.22–1.85)	*p* < 0.001
	Sex (ref.: female)	0.90 (0.84–0.95)	*p* < 0.001	0.93 (0.81–1.06)	*p* = 0.136	0.95 (0.77–1.16)	*p* = 0.476
	CCI	1.24 (1.20–1.28)	*p* < 0.001	1.11 (1.04–1.19)	*p* < 0.001	1.25 (1.14–1.38)	*p* < 0.001
	HTN	1.09 (1.01–1.18)	*p* = 0.004	0.96 (0.82–1.13)	*p* = 0.536	0.92 (0.70–1.20)	*p* = 0.406
	HLD	0.93 (0.86–1.01)	*p* = 0.025	0.91 (0.79–1.05)	*p* = 0.092	1.03 (0.82–1.30)	*p* = 0.716
Other than kidney	Group	1.17 (1.10–1.25)	*p* < 0.001	1.53 (1.33–1.77)	*p* < 0.001	1.27 (1.02–1.58)	*p* = 0.006
	Sex (ref.: female)	1.85 (1.74–1.97)	*p* < 0.001	1.76 (1.53–2.03)	*p* < 0.001	1.76 (1.41–2.21)	*p* < 0.001
	CCI	1.26 (1.22–1.31)	*p* < 0.001	1.15 (1.07–1.23)	*p* < 0.001	1.21 (1.08–1.34)	*p* < 0.001
	HTN	1.04 (0.96–1.13)	*p* = 0.171	0.99 (0.84–1.16)	*p* = 0.859	1.05 (0.79–1.38)	*p* = 0.669
	HLD	0.88 (0.81–0.96)	*p* < 0.001	0.94 (0.81–1.10)	*p* = 0.317	0.95 (0.74–1.21)	*p* = 0.575
**Eye**	Group	0.99 (0.70–1.39)	*p* = 0.924	1.28 (0.55–2.96)	*p* = 0.452	0.38 (0.08–1.68)	*p* = 0.093
	Sex (ref.: female)	1.37 (0.99–1.88)	*p* = 0.010	1.39 (0.61–3.15)	*p* = 0.303	1.08 (0.30–3.92)	*p* = 0.881
	CCI	1.25 (1.05–1.48)	*p* < 0.001	1.07 (0.69–1.66)	*p* = 0.678	1.26 (0.69–2.30)	*p* = 0.323
	HTN	0.97 (0.63–1.48)	*p* = 0.841	0.92 (0.36–2.39)	*p* = 0.830	1.08 (0.23–5.02)	*p* = 0.898
	HLD	1.05 (0.68–1.61)	*p* = 0.780	1.27 (0.54–2.96)	*p* = 0.474	1.32 (0.34–5.06)	*p* = 0.596
**Brain and other central nervous system**	Group	1.31 (1.17–1.48)	*p* < 0.001	1.22 (0.93–1.60)	*p* = 0.059	1.05 (0.67–1.67)	*p* = 0.767
	Sex (ref.: female)	0.96 (0.86–1.07)	*p* = 0.284	1.02 (0.79–1.33)	*p* = 0.819	1.04 (0.66–1.64)	*p* = 0.840
	CCI	1.14 (1.07–1.22)	*p* < 0.001	1.08 (0.94–1.25)	*p* = 0.174	1.10 (0.85–1.41)	*p* = 0.357
	HTN	1.01 (0.87–1.16)	*p* = 0.915	0.74 (0.53–1.02)	*p* = 0.015	0.75 (0.40–1.43)	*p* = 0.254
	HLD	0.83 (0.71–0.96)	*p* = 0.001	0.95 (0.72–1.27)	*p* = 0.664	0.98 (0.59–1.63)	*p* = 0.916
**Lymph and hematopoietic**	Group	1.19 (1.13–1.26)	*p* < 0.001	1.44 (1.27–1.64)	*p* < 0.001	1.11 (0.91–1.36)	*p* = 0.186
	Sex (ref.: female)	1.11 (1.05–1.17)	*p* < 0.001	1.18 (1.04–1.33)	*p* < 0.001	1.06 (0.87–1.29)	*p* = 0.472
	CCI	1.24 (1.20–1.28)	*p* < 0.001	1.07 (0.99–1.14)	*p* = 0.012	1.02 (0.91–1.15)	*p* = 0.652
	HTN	0.98 (0.91–1.05)	*p* = 0.364	0.92 (0.80–1.07)	*p* = 0.165	1.24 (0.97–1.59)	*p* = 0.021
	HLD	0.76 (0.71–0.82)	*p* < 0.001	0.80 (0.70–0.92)	*p* < 0.001	0.68 (0.53–0.86)	*p* < 0.001

Groups: 1. Diabetes vs. non-diabetes: diabetes (reference: non-diabetes); 2. diabetes w/DR vs. diabetes w/o DR: diabetes w/DR (reference: diabetes w/o DR); 3. PDR vs. NPDR: PDR (reference: NPDR)

### Diabetes with DR versus diabetes without DR

During a follow-up period of 12 years, the overall mean annual incidence of total cancer was significantly higher in diabetes patients with DR than in diabetes patients without DR (1494.33 per 100,000 person-years vs. 1151.51 per 100,000 person-years; incidence ratio, 1.32) (Table 2).

In the multivariate survival analysis, diabetes with DR was independently associated with an increased risk of subsequent total cancer development (HR, 1.31; 99% CI: 1.28–1.34; *p* < 0.001). Males also had a moderately higher incidence of subsequent total cancer (HR, 1.25; 99% CI: 1.23–1.28; *p* < 0.001), whereas hypertension and hyperlipidemia did not. Regarding cancer sites, patients with DR showed a significantly increased risk of subsequent liver, mesothelial and soft tissue, and urinary tract cancers. We also observed a moderately increased risk of lip, oral cavity and pharynx, stomach, colon, pancreas, respiratory and intrathoracic organs, skin, female breast, and lymph and hematopoietic cancers. Similarly, patients with DR showed a trend toward an increased risk of subsequent esophageal, gallbladder, bone and articular cartilage, male genitalia, and eye, but the increase did attain a statistically significant difference in the multivariate analysis (Table 3).

### Development of cancer in different stages of DR

The overall mean annual incidence of total cancer was higher in PDR patients than in NPDR patients (1464.64 per 100,000 person-years vs. 1329.56 per 100,000 person-years; incidence ratio, 1.13). Meanwhile, multivariate analysis showed an increased risk of subsequent total cancer development (HR, 1.13; 99% CI: 1.10–1.17; *p* < 0.001) in PDR patients than in NPDR patients.

Regarding the site of cancer, PDR patients showed a moderately increased risk of stomach, liver, female genital, and urinary tract cancer and a borderline significantly increased risk of colon cancer compared to NPDR patients. Similarly, patients with PDR showed a trend toward an increased risk of subsequent cancers of the lip, oral cavity and pharynx, gallbladder, respiratory and intrathoracic organs, bone and articular cartilage, skin, and lymph and hematopoietic cancer, but the increase did attain a statistically significant difference in the multivariate analysis. In contrast, PDR patients showed a decreased risk of male genital cancer compared with NPDR patients (Table 3).

## Discussion

In this study, patients with diabetes were associated with a 20% higher risk of the subsequent development of total cancer incidence compared to patients without diabetes. Notably, we firstly observed that patients with DR had a 32% higher cancer incidence than those without. Furthermore, patients with PDR have a 13% higher risk of cancer in comparison to patients with NPDR. Our study encompasses a nationwide cohort, and the findings contribute to the body of evidence on the relationship between diabetes and cancer risk, confirming prior research on the association between diabetes and cancer risk [12–18]. DR is the most common microvascular complication in patients with diabetes. The propensity to develop DR is directly proportional to patient age, diabetes duration, and poor glycemic control [2]. A meta-analysis including nineteen studies that compared persons with high versus low levels of serum glucose (cut-off > 6.1 mmol/L) showed a positive association between serum glucose and risk of cancer with a pooled RR of 1.32 (95% CI: 1.20–1.45) [19]. Furthermore, sudden variations of blood glucose may play an important role in DR; therefore, glycemic variability (GV) may be useful in predicting complications of diabetes such as DR [20]. Our findings are aligned with the results of a recent prospective cohort study that included 15,286 participants, which indicated that high GV was associated with increased risk of all-site, breast, liver cancer, and cancer-specific death in diabetes [21].

The exact link between diabetes and cancer development remains unclear. Although the analysis of claims data is not designed with biological conclusions in mind, these results raise the hypothesis that DR and cancer may share some possible similar pathogenic features. DR patients have significantly higher levels of serum vascular endothelial growth factor (VEGF) and angiopoietin-2 (Ang-2) than non-DR individuals [22, 23]. Interestingly, both tumorigenesis and DR involve VEGF- and Ang-2-mediated pathways, and pharmaceutical agents targeting these factors have been effective in treating both diseases [24–26]. Moreover, VEGF and Ang-2 promote endothelial cell expression of intercellular adhesion molecule 1, leading to leukocyte activation and cytokine release, thereby causing further increases in VEGF expression and amplifying the inflammatory response [22, 27].

In addition to VEGF and Ang-2, several pathophysiological features have been observed in DR and cancer. First, pericyte loss is the earliest clinical sign of DR; the possible mechanisms linking pericyte apoptosis in DR include increased oxidative stress and nuclear factor-κB (NF-kB) activation [28]. Similarly, pericytes are also implicated as mediators of several processes associated with cancer pathophysiology, including tumor angiogenesis and metastasis [29]. Additionally, NF-κB is the most important molecule linking chronic inflammation to cancer; its activation occurs in cancer cells and tumor microenvironments in most solid cancers and hematopoietic malignancies [30]. Furthermore, platelet-derived growth factors (PDGFs) are growth factors that regulate cell growth and division. Increased PDGF levels, which are the main pathological characteristic of DR, especially the impairment of endothelial migration and proliferation by the inflammatory and angiogenic effects of PDGFs [31]. Intriguingly, PDGF signaling overactivity is associated with the development of numerous types of malignancies [32].

Systemic inflammation is an intrinsic response to diabetes and can promote or increase the risk of many different cancers, including liver, pancreatic, colon, breast, and other malignancies [33]. Several inflammatory mediators play roles in both DR and cancer. Our results revealed that patients with diabetes tended to have a greater cancer risk than their matched controls, and this trend intensified when DR developed. DR development refers to the breakdown of the blood-retinal barrier. Increasing evidence supports the role of proinflammatory cytokines, chemokines, and other inflammatory mediators in the pathogenesis of DR [34–37], leading to persistent low-grade inflammation. The inflammatory mediators released in DR may further trigger cancer pathogenesis, thereby increasing the likelihood of cancer.

The natural history of DR has been divided into two stages based on the proliferative status of the retinal vasculature: early NPDR and advanced PDR; our findings showed that patients with PDR had a higher overall mean annual cancer incidence than those with NPDR. PDR patients exhibit significantly elevated levels of serum interleukin (IL)-1β, tumor necrosis factor α, IL-6, VEGF, and matrix metalloproteinases (MMPs) than NPDR patients [38, 39]. Several studies have demonstrated a positive correlation between MMPs expression and the invasive and metastatic potential of malignant tumor [40]. Furthermore, transforming growth factor ꞵ1 (TGFꞵ1) is a pro-inflammatory cytokine implicated in the pathogenesis of DR, particularly in the late phase of the disease. TGFβ released and activated within the tumor microenvironment promotes cancer progression. Enhanced TGFβ signaling promotes cancer cell invasion, dissemination, and suppresses the sensitivity to anticancer drugs [41]. Our study findings are also supported by the fact that PDR patients have more severe pathology and inflammation than NPDR patients. In addition, cancer-associated retinopathy is a rare paraneoplastic disorder with loss of visual acuity caused by circulating antibodies formed against the retinal proteins in the presence of systemic cancer [42]. Since our patients had DR first and subsequently developed cancer, these two types of retinopathy have different etiological bases.

Except for female breast cancer, we found a significantly lower incidence of all-site cancer in patients with hyperlipidemia than in those without hyperlipidemia. Most epidemiological studies have reported inconsistent results regarding the association between hyperlipidemia and cancer incidence [43–53]. Cancer is known to have a protean physiological effect, which might include metabolic depression of blood cholesterol [54] or competing risks, and patients showing high total serum cholesterol (TSC) levels are more likely to be censored owing to cardiovascular mortality before they are diagnosed with cancer [55]. However, some studies found inverse associations with a time lag of ≥ 4 years between baseline cholesterol level and cancer diagnosis [48, 55, 56]; thus, the possibility of a direct effect of cholesterol on cancer still cannot be completely ruled out. In our study, hyperlipidemia was positively associated with breast cancer in women. Previous animal models have implied that increased plasma cholesterol levels might accelerate breast cancer development and exacerbate their aggressiveness [57]. Our results are also consistent with findings from a prospective large longitudinal cohort study in Korean adults [50], which showed that high TSC levels were positively associated with breast cancer risk in women.

This study has several strengths. First, the NHIRD contains all claims data recorded electronically, ensuring accuracy and avoiding recall biases. Second, data from the NHIRD provide population-based and representative claims information for insured people in Taiwan and reduce the likelihood of selection bias. Third, the large dataset size and longitudinal study design provided considerable statistical power, enabling the effective detection of differences between the cancer and control cohorts. This type of longitudinal cohort study has advantages over cross-sectional or case–control studies because the design allows the researchers to examine the natural course of cancer development over an extended period of time. However, some shortcomings of this type of design such as death as the competing risk for the event should be considered.

## Study limitations

First, the NHIRD is an administrative database lacking laboratory results, such as HbA1c, and it cannot differentiate between the subtypes of hyperlipidemia (hypercholesterolemia, hypertriglyceridemia, or both in combined hyperlipidemia). Some important risk factors for cancer are not available in the NHIRD, such as education level, drinking and smoking habits, body mass index, physical activity, and family history of cancer, which might have confounded our results. Second, although the current retrospective cohort study was more efficient than a prospective study, some potential risk factors could not be obtained owing to the retrospective nature of the study. Third, early cancer might have been asymptomatic, and individuals with early cancer might have been undiagnosed, which could have led to misclassification bias. This non-differential misclassification could bias our results toward the null hypothesis and dilute the real difference in cancer incidence between the two cohorts. Fourth, early-stage diabetes might also have been underdiagnosed, which could have resulted in group misclassification. Fifth, this study lacked information on the patients’ use of cholesterol-lowering drugs. However, previous studies have not suggested a strong link between drug use and the incidence of cancer [58, 59]. Sixth, most of our study participants were ethnically Chinese from Taiwan, which might affect the generalizability of our results to other ethnic groups. Finally, this epidemiological study only supports the concept that there is a correlation between diabetes and tumorigenesis. Direct evidence to prove the causal relationship between these two diseases is not feasible to obtain in human studies considering the long follow-up period to identify the event (cancer). Perhaps, findings from relevant animal models may provide novel evidence to support this relationship. Analyzing serum biomarkers related to cancer development may help to provide some indirect evidence to support the concept. However, NHIRD does not contain laboratory data from patients, and clinical tests that analyze patients’ samples are not part of the scope of this study.

## Conclusions

This nationwide population-based study provides evidence reinforcing an association between diabetes and the overall cancer incidence. To our knowledge, we first explored the association between DR and cancer. The result contributes novel insights by unveiling that patient with DR were at a significantly greater risk of subsequent cancer development at specific sites than their matched controls. These results raise the possibility that diabetes and DR may share common pathogenic features with cancer, and strict blood glucose control to prevent DR in patients with diabetes may further reduce cancer development. Further studies are required to better understand the underlying processes.

## Data Availability

Data are available from the National Health Insurance Research Database (NHIRD) published by the Taiwan National Health Insurance (NHI) Bureau. Due to the legal restrictions imposed by the government of Taiwan in relation to the “Personal Information Protection Act,” data cannot be made publicly available. Requests for data can be sent as formal proposals to the NHIRD (http://nhird.nhri.org.tw).

## Abbreviations

Ang-2: Angiopoietin-2
CCI: Charlson index comorbidity
CI: Confidence intervals
DR: Diabetic retinopathy
GV: Glycemic variability
HR: Hazard ratios
ICD-9-CM: International Classification of Diseases, 9th Revision, Clinical Modification
ICD-10-CM: International Classification of Diseases, 10th Revision, Clinical Modification
IL: Interleukin
MMPs: Matrix metalloproteinases
NDR: National Death Registry
NF-κB: Nuclear factor-κB
NHIRD: National Health Insurance Research Database
NPDR: Non-proliferative diabetic retinopathy
PDGFs: Platelet-derived growth factors
PDR: Proliferative diabetic retinopathy
SMD: Standardized mean difference
TCR: Taiwan Cancer Registry
TGF β: Transforming growth factor β
TSC: Total serum cholesterol
VEGF: Vascular endothelial growth factor
